# Facial asymmetry: virtual planning to optimize treatment predictability and aesthetic results

**DOI:** 10.1590/2177-6709.23.6.080-089.bbo

**Published:** 2018

**Authors:** Hélio Henrique de Araújo Brito, Carolina Morsani Mordente

**Affiliations:** 1 Pontifícia Universidade Católica de Minas Gerais, Programa de Pós-graduação em Odontologia, Departamento de Ortodontia (Belo Horizonte/MG, Brazil).

**Keywords:** Facial asymmetry, Orthognathic surgery, Virtual planning

## Abstract

Facial asymmetry is a condition that compromises function and social interactions and, consequently, the quality of life. Orthodontic-surgical treatment may be indicated to achieve a stable occlusion and significant improvement in facial aesthetics. The virtual planning of the maxillary, mandibular and chin movements can be done prior to surgery. These movements can be successfully performed with the use of prototyped guides obtained from virtual planning. The aim of this article is to show the state of the art of treatments of facial asymmetries, and emphasize how important is the multi-disciplinary approach to achieve predictable aesthetic and functionally stable results in a patient with facial asymmetry and chin protrusion.

## Resumo

A assimetria facial é uma condição capaz de comprometer a função oclusal e as interações sociais e, consequentemente, a qualidade de vida dos indivíduos. Nessas condições, para se obter oclusão estável e melhora significativa na estética facial, o tratamento ortodôntico-cirúrgico pode estar indicado. A simulação virtual da cirurgia permite planejar de forma adequada, e antecipada, os movimentos cirúrgicos a serem efetuados na maxila, mandíbula e mento. Esses movimentos são, então, realizados com sucesso graças ao uso de guias prototipados obtidos a partir do planejamento virtual. Assim, os objetivos do presente artigo consistem em relatar o estado da arte no planejamento virtual do tratamento de um paciente com assimetria facial e protrusão do mento, e enfatizar a importância da abordagem multidisciplinar para se atingir resultados estéticos previsíveis e funcionalmente estáveis.

## INTRODUCTION

Facial asymmetry is a craniofacial deformity that is frequently reported as a chief complaint of orthodontic patients. It may compromise the social interactions and thus the quality of life of the affected individuals.^1^ ^,^ ^2^

The lower third of the face is more frequently affected than the middle and upper thirds, the latter being the least affected. Generally, when there is asymmetry in the middle and upper thirds, mandibular asymmetry is also present.^1^ Asymmetries of the lower third of the face may be mandibular, isolated or maxillomandibular.^3^

It may have a specific cause but usually is the result of complex interactions of multiple factors that interfere with facial growth and development. Proffit et al^4^ divide the etiological factors of dentofacial deformities into three main groups: 1) specific causes, such as syndromes, congenital anomalies, and growth and developmental disorders; 2) hereditary factors; (3) environmental influences, such as soft tissue pressure, bite force, and respiratory influences.^4^

Bishara et al^5^ classified the craniofacial asymmetries, according to the structures involved, in: skeletal, muscular or functional. However, differential growth between the two sides of the face can occur as an adaptive response to functional deviations, causing a significant remodeling of the condyle and mandibular fossae, that may transform a functional asymmetry into a skeletal asymmetry.^1^ ^,^ ^6^ This condition is often associated with a Class III malocclusion.^6^

Obwegeser^7^ classified the asymmetries as type I, or hemimandibular hyperplasia; type II, or hemimandibular elongation; and type III, hybrid form of types I and II. Type I is characterized by the three-dimensional increase of mandibular volume, not resulting in a deviation of the symphysis or crossbite. Type II is characterized by the elongation of the entire jaw unilaterally; therefore, the chin deviates to the opposite side and a crossbite is developed. Type III is characterized by the elongation and increased volume of the affected side of the mandible, with the chin deviated to the opposite side. 

The precise diagnosis of facial asymmetries in the frontal plane, measuring the differences between the right and left sides, and the vertical asymmetries, are crucial for the development of a treatment plan capable of promoting harmonic and stable results. Accuracy is essential and the computed tomography represents a valuable tool.^8^ It can reproduce all hard tissues of the face, three-dimensionally revealing the entire craniofacial complex.^9^

Orthognathic surgery planning has considerably evolved over the last decades. Initially, it was performed with cephalometric and facial analysis, and plaster casts of the patient’s dental arches mounted on an articulator for the manufacture of surgical splints. Planning has evolved from the use of two-dimensional computer programs to more modern computer-based planning techniques.^8^

This new technology integrates the diagnosis, planning and surgical intervention using a software that performs patient’s analysis in three dimensions. The virtual platform is then used in the preoperative stage, to plan the surgical movements.^10^ Then, they are transferred from the virtual planning to the surgical procedure using prototyped surgical splints.^10^

Virtual planning represents an important tool to correct facial asymmetries, allowing greater accuracy in the treatment of these patients, both from the orthodontic and surgical perspectives.^11^ Some authors affirm that computerized planning increases the effectiveness of orthognathic surgery, providing more precise osteotomies than with classic planning.^12^ The use of this modern planning technology for surgical treatments contributes to better and more predictable results.^13^

Thus, the aim of this article was to show the state of the art of treatments for facial asymmetries, and emphasize how important is the multi-disciplinary approach to achieve predictable aesthetic and functionally stable results in a patient with facial asymmetry and chin protrusion. 

## CASE REPORT 

**Figure 1:**
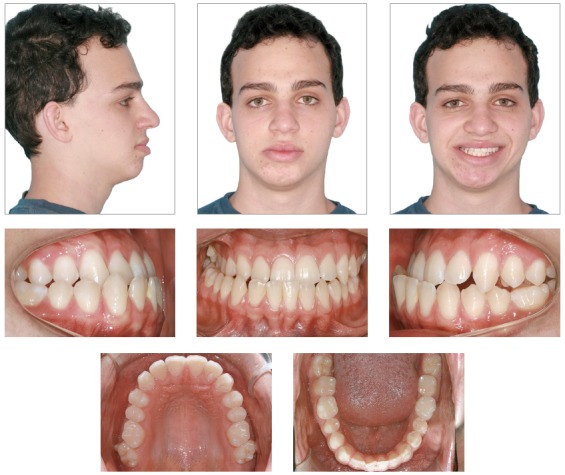
Extra and intraoral initial photos.

The extraoral exam revealed an asymmetrical face, with the chin deviated to the right side and maxillary occlusal plane canted. As suggested by Obwegeser’s^7^ classification, the patient presented a Type II asymmetry, characterized by an elongation of the mandible on the left side, which results in chin deviation to the right side. Moreover, there was no increase in volume on the affected side (Fig 1). 

The lips had a passive seal and the lower third of the face was decreased in relation to the others. The profile was straight and associated with a protrusion of the lower lip (7 mm ahead of the Steiner’s S-line). The aesthetics of his smile was compromised due to the minor exposure of the maxillary posterior teeth on the right side, and the excessive exposure of the mandibular teeth, especially of the right side. 

**Figure 2:**
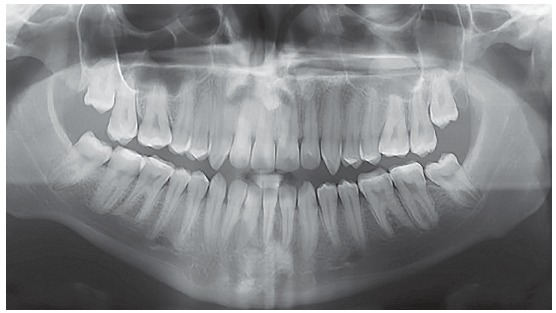
Initial panoramic radiograph.

The intraoral exam revealed molars and canines in a Class III relationship on both sides. He also had a posterior crossbite on the right side, as well as an anterior crossbite (Fig 2). The upper midline was coincident to the facial midline and the lower midline, deviated 4 mm to the right. Both dental arches presented adequate forms, with small diastemas on the upper and lower arches, and mild rotation of the lower right central incisor. Functionally, lateral and anterior occlusal guidances were absent. 

**Figure 3:**
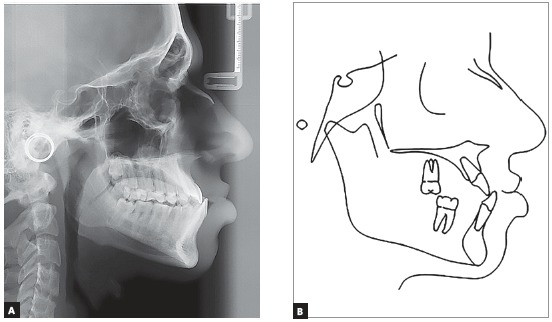
A) Teleradiography; B) initial cephalometric tracing.

The panoramic radiograph showed that all teeth present with no significant periodontal alterations. The initial cephalogram (Fig 3) revealed an ANB angle of -1^o^ due to maxillary deficiency (SNA = 80^o^) and a mild mandibular protrusion (SNB = 81^o^), with a Angle of convexity of -4^o^. The patient did not present any vertical alteration. The FMA, Y-axis and SN-GoGn were equal to 13^o^, 48^o^ and 32^o^, respectively. The maxillary and mandibular incisors were flared (1.NA = 29^o^; 1-NA = 6 mm; 1.NB = 27^o^; 1-NB = 6.5 mm).

## TREATMENT PLAN 

The treatment objectives were to correct the skeletal discrepancies and: 1) dental compensations created by malocclusion; 2) posterior crossbite; 3) vertical maxillary asymmetry; 4) mandibular asymmetry; 5) lower lip protrusion; as well as achieve normal occlusal relationships, with improvement of patient’s facial asymmetry and aesthetics. Therefore, an orthodontic treatment associated with orthognathic surgery was proposed. Due to the complexity of the malocclusion and its skeletal component in all planes of the space, it was not considered any other treatment alternative. The patient and his parents agreed with the treatment plan proposed. 

Initially, the patient was referred to the bucomaxillofacial surgeon for a first appointment, preliminary orientations and decision about the extraction of the third molars. After that, fixed appliances were installed and dental leveling and alignment were performed with a routine archwire sequence. A 0.017*x*0.025-in stainless steel archwire with loops was used to close the spaces. 

**Figure 4:**
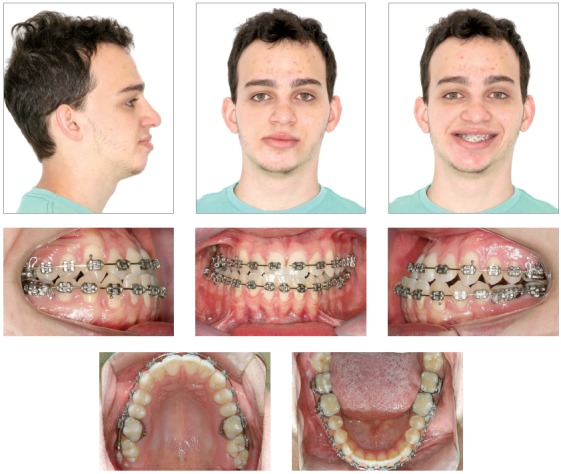
Extra and intraoral intermediate photos.

**Figure 5:**
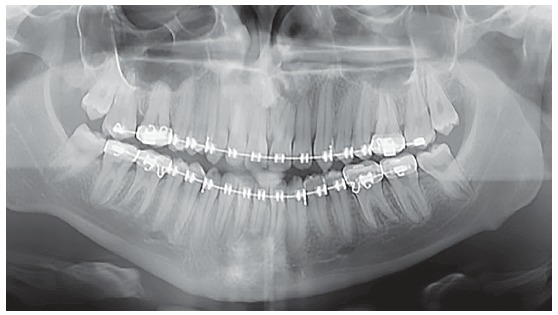
Intermediate panoramic radiograph.

**Figure 6:**
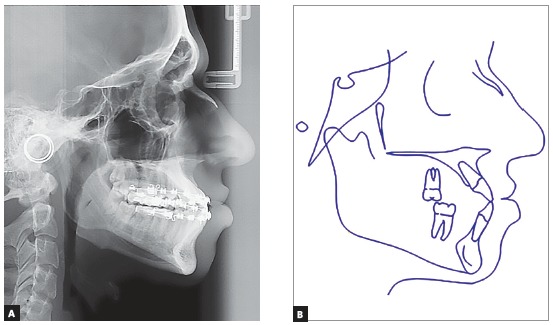
A) Teleradiography; (B) intermediate cephalometric tracing.

**Figure 7:**
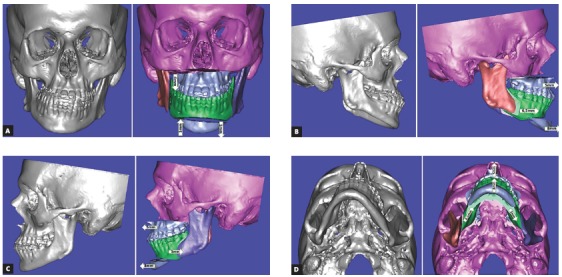
Virtual surgical planning of bone movements in frontal (A), right (B), left (C) and lower (D) views.

When the study plaster casts showed good coordination and intercuspation (Figs 4 to 6) between the arches, it was requested a computerized tomography of the head for virtual surgical planning. It revealed to be necessary a Le Fort I osteotomy with 5 mm of advance and 4 mm of inferior repositioning of the posterior segment on the right side. For the mandible, it would be necessary 6.5 mm of advance on the right side, 3.0 mm of setback on the left side; and, for the chin, 8 mm of advance, 1 mm of inferior repositioning of the right side and 1 mm of superior repositioning on the left side. It was suggested for the patient to undergo rhinoplasty, since some enlargement is expected with the maxillary advance (Fig 7). 

Hooks were soldered on 0.019*x*0.026-in stainless steel archwire, and the patient was referred for the orthognathic surgery. A rhinoplasty was performed immediately after surgery, and both occurred with no complications. The pre-surgical orthodontic phase permitted an adequate intercuspation after surgery. During the post-surgery orthodontic phase, the patient used vertical intermaxillary elastics and some brackets were rebonded to obtain radicular parallelism. After debonding, a wraparound retainer on the upper arch and a modified spring retainer on the lower arch, both removable, were installed.

## TREATMENT RESULTS

**Figure 8:**
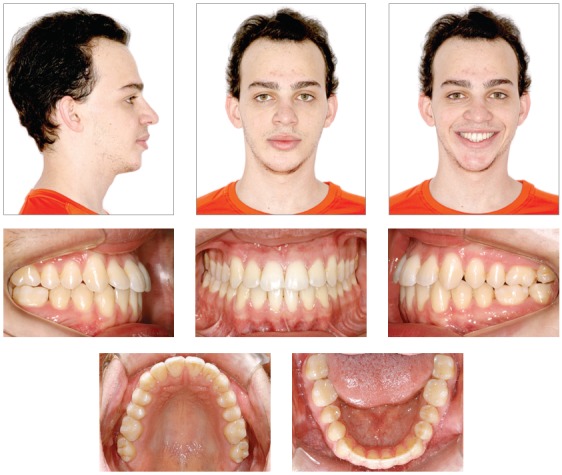
Final extraoral and intraoral photos.

A significant improvement was seen in facial asymmetry and mandibular deviation. A pleasant facial smile was obtained with the correction of the cant of the upper occlusal plane, increase in the upper right teeth exposure, and decrease in the lower teeth exposure, especially on the right side. The facial profile remained straight, with reduction of the lower lip protrusion. It is possible to observe that the width of the base of the nose was held, when the face is compared before and after surgery (Fig 8). 

Intraoral records demonstrated good occlusal relationships, with canines and molars in Class I and good intercuspation, which was possible due to the coordination of the arches and buccal torque of the root of the lower posterior teeth. The dental midlines became coincident, and normal overbite and overjet were present (Fig 8). Because of the improvement in occlusion, the patient presented appropriate lateral and anterior occlusal guidances. 

**Figure 9:**
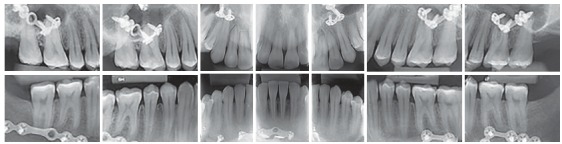
Final periapical radiographs.

**Figure 10:**
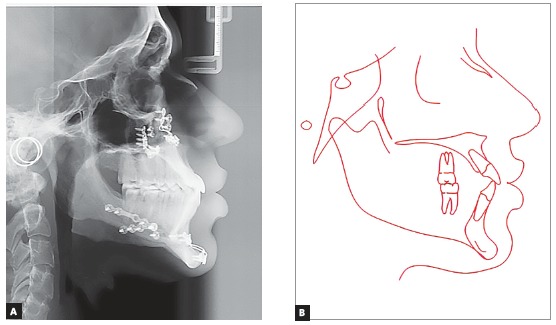
A) Teleradiography; (B) final cephalometric tracing.

**Figure 11:**
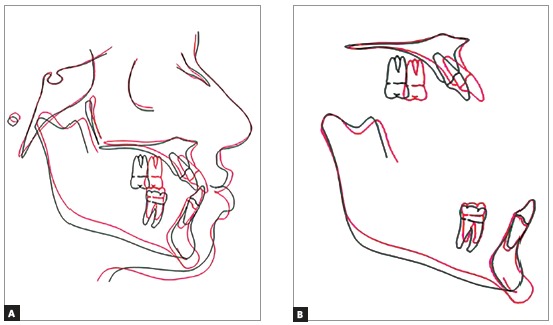
Total (A) and partial (B) superimpositions of the initial (black) and final (red) cephalometric tracings.

Cephalometric superimpositions showed a significant improvement in the position of the maxilla, due to the advance and selective impaction of the posterior segment. The mandible only had an expected small posterior displacement, since the main objective of the mandibular surgery was to rotate it, and not set it back. These modifications allowed an improvement in the sagittal skeletal disharmony, with an increase of the ANB angle from -1^o^ to 4^o^. The increase of the SN.GoGn angle was due to the chin advance of 8 mm, which interfered on the Gnation’s vertical position. The upper and lower incisors had their proclination increased, and the lower molars were extruded at the end of treatment (Figs 9 to 12). 

Evaluating the total cephalometric tracings superimposition and the facial contour changes, it is possible to observe that in spite of the significant advancement of the maxilla, the nose did not have its morphology altered, both vertically and horizontally. The chin advancement and inferior positioning were accompanied by the soft tissue, giving a more defined aspect to the chin and lower lip region. 

**Table 1:** Initial (A) and final (B) cephalometric values

	Measurements		Normal	A	A1	B	A/B diff.
Skeletal pattern	SNA	(Steiner)	82°	80°	81°	84°	4
	SNB	(Steiner)	80°	81°	81°	80°	1
	ANB	(Steiner)	2°	-1°	0°	+4°	5
	Wits	(Jacobson)	♀ 0 ±2 mm ♂ 1 ±2 mm	-3 mm	- 1.5 mm	+3 mm	6
	Angle of convexity	(Downs)	0°	-4°	- 2°	+2°	6
	Y-axis	(Downs)	59°	48°	48°	48°	0
	Facial angle	(Downs)	87°	97°	98°	100°	3
	SN-GoGn	(Steiner)	32°	28°	28°	32°	4
	FMA	(Tweed)	25°	13°	13°	18°	5
Dental pattern	IMPA	(Tweed)	90°	96°	97°	96°	0
	1.NA (degrees)	(Steiner)	22°	29°	35°	29°	0
	1-NA (mm)	(Steiner)	4 mm	6 mm	7 mm	7 mm	1
	1.NB (degrees)	(Steiner)	25°	27°	27°	30°	3
	1-NB (mm)	(Steiner)	4 mm	6.5 mm	7 mm	7 mm	0.5
	- Interincisal angle	(Downs)	130°	125°	117°	117°	8
	1-APo	(Ricketts)	1 mm	8 mm	7 mm	3 mm	5
Profile	Upper lip - S-line	(Steiner)	0 mm	0.5 mm	0 mm	1 mm	0.5
	Lower lip - S-line	(Steiner)	0 mm	7 mm	6 mm	3 mm	4

## DISCUSSION

Orthognathic surgery may be required as part of the treatment of facial asymmetries, which are challenging for orthodontists, surgeons, as well as for patients, due to their impact on the psychological welfare, self-esteem, and quality of life.^14^ Therefore, careful diagnosis and appropriate treatment plans can ensure predictable results in the treatment of these dentofacial deformities.^15^

Planning orthognathic surgeries used to be done with simulations based on bidimensional lateral radiographs, facial analysis, and plaster casts mounted on an articulator. Nowadays, planning evolved from the cephalometric tracing in two dimensions to a modern technology of virtual planning.^8^ ^,^ ^10^ This new alternative has become an important tool to plan facial asymmetry treatments, and the 3D platform is normally used at a further preparatory step, which aims to have surgical movements planned. It allows for greater accuracy in asymmetrical patient’s treatment, from both surgical and orthodontic perspectives.^11^ Some authors have reported that virtual planning increases the effectiveness as orthognathic surgery, with more accurate osteotomies than with the classic planning.^12^ ^,^ ^13^ The use of this modern digital technology contributed to the more predictable and good results obtained in this patient. 

Le Fort I osteotomies with maxillary advance and/or impaction can cause a widening of the nose, as well as sagittal and vertical changes.^16^ These undesirable side effects can be counteracted by a rhinoplasty performed at the same time of the orthognathic surgery.^17^ This is possible due to the recent progress in surgical techniques. It is also very important to explain the patients the reasons to perform these two procedures at the same time. 

The total superimposition of cephalometric tracings revealed that, in spite of the 5 mm of maxillary advance, no sagittal change was observed in the nose. Vertically, only a small change was present in the region of the columella, which did not significantly interfere in the facial aesthetic result, confirming the importance of rhinoplasty. This procedure was able to counteract the side effects of the orthognathic surgery. The changes in the lower third of the face revealed the upper lip was positioned more inferiorly, had an increase in thickness and was advanced approximately half of the amount of the maxillary advancement. The lower lip was retracted and positioned more inferiorly. The chin was advanced 8 mm and displaced inferiorly. The soft tissue accompanied this movement, but in a smaller amount. All these soft tissue changes in the lower third made the contour of the patient’s face more harmonic and pleasant.

## CONCLUSIONS

» The virtual planning allowed the precise reproduction of the previously defined bone movements, during the actual surgical procedures.
» The multi-disciplinary approach involving orthodontics, orthognathic and plastic surgery allowed the improvement of the facial esthetics and an occlusion with correct intercuspation, functionally efficient and stable.
